# A promising route to neuromorphic vision

**DOI:** 10.1093/nsr/nwaa182

**Published:** 2020-08-21

**Authors:** Leon Chua

**Affiliations:** Electrical Engineering and Computer Sciences Department, University of California, USA

The human vision system has a very strong capability in perceiving surroundings. Such visual perception starts in the retina, which receives and preprocesses the visual input in the form of light, and ends up with high-level processing in the visual cortex of the brain (Fig. 1a). As such, we can understand what the visual inputs represent while consuming little energy. It has been a long-sought dream for human beings to build a powerful and energy-efficient intelligent vision system that has a superior ability, similar to the human brain. Computer vision, as a similar model to the human brain, aims at viewing, processing and understanding images in the same way as human beings, and has become one major technological advancement in building intelligent machines [1]. However, the mainstream technology for computer vision is based on algorithms running on a von Neumann architecture computer, and cannot emulate the hierarchical organizations and biological functions of the human vision system. In particular, traditional computer vision in conjunction with the conventional charge-coupled device (CCD) and complementary metal-oxide-semiconductor (CMOS) image sensors suffers from challenges in latency and power consumption as a high volume of redundant visual information is sensed and then has to be processed.

**Figure 1.**
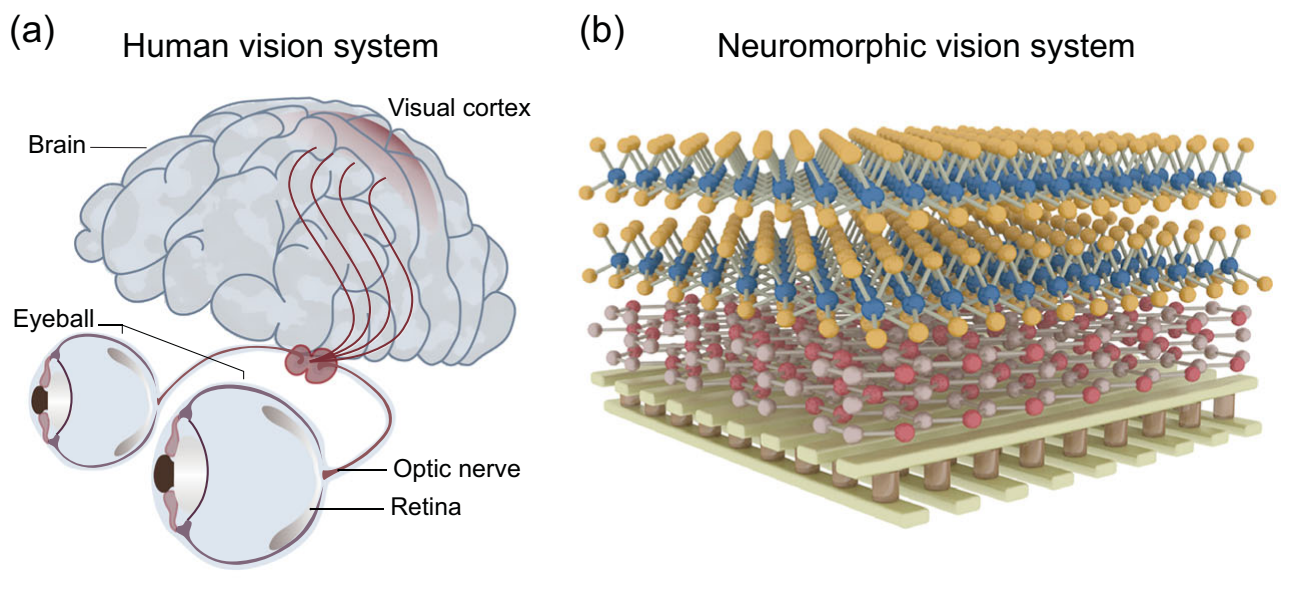
Human vision system (a) and neuromorphic vision system (b) composed of crossbar array and van der Waals heterostructure materials.

A recent work [2] on the development of the neuromorphic visual system attempted to replicate the human's capability of visual perception and represents a significant advancement towards the creation of intelligent machines. This interesting work led by Shi-Jun Liang and Feng Miao at Nanjing University reports development of a brain-inspired visual perception system by networking a retinomorphic sensor and a memristive crossbar, in an effort to overcome the challenges faced with conventional computer vision. Using van der Waals heterostructure and a memristive crossbar to emulate the retina and visual cortex of the human vision system, respectively, as schematically shown in Fig. 1b, Liang and Miao *et al.* for the first time built up a neuromorphic vision system with hierarchical organizations and biological functions similar to that of the human brain. The retinomorphic sensor based on van der Waals heterostructure not only mimics the hierarchical structure of the human retina but also replicates its biological functionalities in image sensing and early processing. By networking with a large-scale memristive crossbar, they demonstrated that the early processing occurring in the retinomorphic sensor allows for drastically improving pattern recognition, which can be modelled by cellular neural networks [3,4]. Furthermore, they showed that the proposed architecture also holds great promise in carrying out object tracking tasks.

The work led by Liang and Miao is a major breakthrough in prototype demonstration of a neuromorphic vision system that allows for image sensing and processing as well as perception in the full analog domain. This encouraging work opens up a promising route to be followed in the future for exploring its applications in driverless cars, smart surveillance, intelligent healthcare, etc. We can envisage that this work could stimulate further explorations of more advanced intelligent systems by highly mimicking human beings.

***Conflict of interest statement.*** None declared.

## References

[1] Szeliski R . Computer Vision: Algorithms and Applications. London: Springer Science & Business Media, 2010.

[2] Wang S , Wang CY, Wang P et al. Natl Sci Rev 2021; 8: nwaa172. 10.1093/nsr/nwaa172 PMC8288371 34691573

[3] Chua L . CNN: A Paradigm for Complexity. Singapore: World Scientific, 1998.

[4] Chua L , Roska T. Cellular Neural Networks and Visual Computing: Foundations and Applications. Cambridge: Cambridge University Press, 2002.

